# Inter-trial priming does not affect attentional priority in asymmetric visual search

**DOI:** 10.3389/fpsyg.2014.00957

**Published:** 2014-08-29

**Authors:** Liana Amunts, Amit Yashar, Dominique Lamy

**Affiliations:** The School of Psychology Sciences and The Sagol School of Neuroscience, Tel Aviv UniversityTel Aviv, Israel

**Keywords:** priming of pop-out, inter-trial priming, search asymmetry, preattentive processing, attentional priority allocation, serial search, visual search

## Abstract

Visual search is considerably speeded when the target's characteristics remain constant across successive selections. Here, we investigated whether such inter-trial priming increases the target's attentional priority, by examining whether target repetition reduces search efficiency during serial search. As the study of inter-trial priming requires the target and distractors to exchange roles unpredictably, it has mostly been confined to singleton searches, which typically yield efficient search. We therefore resorted to two singleton searches known to yield relatively inefficient performance, that is, searches in which the target does not pop out. Participants searched for a veridical angry face among neutral ones or vice-versa, either upright or inverted (Experiment 1) or for a Q among Os or vice-versa (Experiment 2). In both experiments, we found substantial intertrial priming that did not improve search efficiency. In addition, intertrial priming was asymmetric and occurred only when the more salient target repeated. We conclude that intertrial priming does not modulate attentional priority allocation and that it occurs in asymmetric search only when the target is characterized by an additional feature that is consciously perceived.

## Introduction

Recent research has demonstrated that attention is directed by past experience: how attention is deployed at a certain moment in time greatly affects how attention will be deployed a moment later. Such effects have most often been demonstrated in the context of visual search, by means of a variety of inter-trial priming effects (e.g., Maljkovic and Nakayama, 1994, 1996; Found and Müller, 1996; Lamy et al., 2008a,c; Yashar and Lamy, 2013). Performance benefits can be very substantial, and reach up to 15% of overall response times (e.g., Lamy et al., 2013).

In the present study we focus on the finding that when observers search for a target with a unique feature on a given dimension among homogenous distractors and the target and distractor features switch unpredictably from trial to trial, responses to the target are faster when the target and distractor features remain the same relative to the preceding trial than when they switch (Maljkovic and Nakayama, 1994; see Kristjánsson and Campana, 2010; Lamy and Kristjánsson, 2013, for reviews). This phenomenon has been generalized to singleton targets defined on various stimulus dimensions and is usually referred to as “Priming of pop-out” (PoP, Maljkovic and Nakayama, 1994). However, it has also been extended to targets that do not pop out, such as targets defined by a unique yet complex feature (e.g., Lamy et al., 2008b) or by a conjunction of features (e.g., Hillstrom, 2000). Therefore, in the remainder of this paper, we use the more general term of “inter-trial priming” to designate the effect of repeating vs. switching the target and distractor features.

### Inter-trial priming and search efficiency

Theories of inter-trial priming suggest that at least part of the effect results from enhanced perceptual processing of the target when it repeats from the previous trial (e.g., Maljkovic and Nakayama, 1994; Becker, 2008; Sigurdardottir et al., 2008; Lamy et al., 2010; Yashar and Lamy, 2010; but see Huang et al., 2004). However, what perceptual processes are speeded by inter-trial priming is a debated issue.

Since Neisser (1967), the distinction between preattentive and attentive perceptual processing has been widely embraced (e.g., Bundesen, 1990; Treisman and Sato, 1990; Wolfe, 1994). Preattentive processing guides attention toward objects of potential interest and is followed by the deployment of focal attention to the locations of high-priority objects (which has been decomposed into more finer-grained sub-stages, e.g., Posner and Petersen, 1990; Folk et al., 2009; Wan and Lleras, 2010; Yashar and Lamy, 2010). Within this framework, some authors have suggested that intertrial priming modulates preattentive processing of the search display. For instance, the initial account suggested by Maljkovic and Nakayama (1994, 1996) stipulates that repetition of the target's feature increases the salience of this feature (and decreases the salience of the distractors' feature) in subsequent trials. Specifically, they suggested that the search-relevant feature of a target selected on a given trial is encoded in a memory trace, which results in a carry-over effect that increases the attention-grabbing capacity of this feature on subsequent trials (see also Becker, 2008; Becker and Horstmann, 2009). Thus, according to this account, repetition of the target feature increases the attentional priority allocated to a target possessing this feature on subsequent trials.

By contrast, others authors proposed that intertrial priming does not affect attentional priority but only subsequent attentive processes (e.g., Yashar and Lamy, 2010, 2011; see also Lleras et al., 2008). Yashar and Lamy (2010) compared the effects of inter-trial priming on search accuracy using brief search displays in a left/right hemifield localization task vs. a fine-discrimination task. They reasoned that if inter-trial priming increases target salience, then feature repetition should benefit performance in both tasks. If instead intertrial priming speeds processes that follow detection of the target, such as shifting attention to its location or engaging attention in it^1^, then improved accuracy on repeated-target trials should be observed only in the discrimination task—which is what they found.

In order to test these accounts against each other more directly, one can investigate whether a target enjoys a competitive advantage over surrounding distractors when its defining feature repeats from the previous trial relative to when it does not. However, when intertrial priming is studied during pop-out search, it is difficult to unambiguously relate priming benefits to increased attentional priority of the repeated feature because in this context, the target is always the most salient object in the display. The outcomes from two different lines of research that circumvent this problem have generated conflicting findings.

Several studies manipulated the presence of a distractor that was more salient than the target and interfered with search. If intertrial priming increases the target's feature salience, then the performance cost associated with the salient distractor's presence should be reduced when the target feature repeats relative to when it switches. While some studies showed that this is indeed the case (e.g., Pinto et al., 2005; Meeter and Olivers, 2006), others reported that intertrial priming does not reduce distractor interference (Lamy et al., 2006; Becker, 2008)^2^. Follow-up experiments suggested a resolution of this discrepancy by demonstrating that the interaction between distractor interference and intertrial priming resulted from strategic factors rather than from increased target salience (Lamy and Yashar, 2008; see Lamy and Kristjánsson, 2013 from a more detailed account). Thus, there is overall only scarce evidence that intertrial priming reduces distractor interference.

Other studies examined inter-trial priming using search tasks in which the target was defined by the conjunction of two features with each distractor sharing one of the target's features. As conjunction search often yields serial search slopes, the prediction is that if intertrial priming increases the target's salience, then search slopes should be reduced when the target repeats. Most of these studies reported significant effects of intertrial repetition on overall RTs but no effect on search efficiency (e.g., Hillstrom, 2000; Koshino, 2001; Kristjánsson et al., 2002; Geyer et al., 2006). Yet, one study (Becker and Horstmann, 2009) reported shallower search slopes for repeated targets. It is noteworthy that by contrast with singleton-search investigations of inter-trial priming, target-feature predictability on any given trial was generally very high in these conjunction-search studies: the possible targets were presented in streaks (e.g., Kristjánsson et al., 2002; Wang et al., 2005), in orderly sequences (e.g., Hillstrom, 2000) or targets and distractors never exchanged roles (e.g., Becker and Horstmann, 2009). Thus, search could be guided by top-down factors to various extents, and such guidance rather than inter-trial priming could explain the few reports of reduced search slopes on repeated-target trials.

The primary goal of the present study was to determine whether intertrial priming modulates attentional priority by investigating whether it reduces search slopes in a search task that cannot be guided by advance knowledge of the potentially repeating feature.

### Inter-trial priming and search asymmetry

An important phenomenon known to affect search efficiency is search asymmetry. A visual search asymmetry is said to occur when given two different stimuli A and B, singleton search is faster and more efficient when A serves as the target and B is used for the background distractors than vice-versa. For example, Treisman and Souther (1985) showed that searching for a Q among Os is easier than searching for an O among Qs. Search asymmetries have also been reported with complex stimuli. One well-documented instance is the face-in-the-crowd effect (e.g., Hansen and Hansen, 1988). The typical finding is that search slopes are shallower when the target is a threat-related face among neutral or positive faces vs. a neutral or positive face among threatening faces (see Eastwood and Smilek, 2005; Horstmann and Bauland, 2006; Horstmann, 2007; Frischen et al., 2008 for extensive reviews).

In a recent study, we reported that search asymmetry in a face-in-the-crowd search manifests not only with regard to overall RTs and search efficiency but also with regard to intertrial priming (Lamy et al., 2008b). Participants had to detect the face displaying a discrepant expression of emotion in an array of four face photographs. On each trial, the target when present was either a neutral face among emotional faces (angry in Experiment 1 or happy in Experiment 2), or an emotional face among neutral faces. Target detection was faster when the target displayed the same emotion on successive trials (although this emotion was displayed by different individuals on consecutive trials). This emotional priming effect occurred for angry and for happy faces, but not for neutral faces. It was entirely abolished when the same faces were inverted instead of upright, suggesting that emotional categories rather than low-level physical feature properties drove the repetition effect.

In addition to determining whether inter-trial priming increases search efficiency, the second objective of the present study was to further investigate the relationship between inter-trial priming and search asymmetry. We examined whether the asymmetric intertrial priming observed with complex stimuli (veridical emotional faces) generalizes to asymmetric searches involving simple features.

## Experiment 1

**Figure 1:**
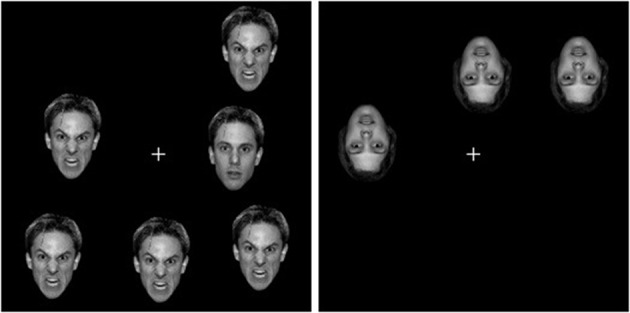
**Sample displays in Experiment 1**. The left-hand panel correspond a neutral target-present trial, with set size 6 and upright faces. The right-hand panel corresponds to a all-neutral target-absent trial, with set size 3 and inverted faces. The stimuli are not drawn to scale.

This experiment was similar to Lamy et al.'s emotional face detection experiment (2008b, Experiment 1) except that the number of items in the display varied instead of being fixed. On each trial, either three or six face pictures displaying the same individual were presented. On target-absent trials, all the faces were identical and on target-present trials, the target was either an angry face among neutral faces or a neutral face among angry faces. Thus, the target and distractors' emotional expressions switched unpredictably from trial to trial. Participants had to detect the presence of a face displaying a discrepant emotional expression. For half of the participants, all faces in a display were presented in their canonical upright orientation, whereas for the remaining half, all faces were inverted (see Figure 1). Note that we used a relatively large pool of different individuals' faces (eight) in order to minimize the chances that emotion-irrelevant features idiosyncratic to the selected stimuli might render angry faces more salient than neutral faces. For the same reason, mean luminance and contrast was equated for the two expressions of each individual.

We expected to replicate the search asymmetry typical of the face-in-the-crowd phenomenon, namely, we expected faster RTs and shallower search slopes for angry than for neutral targets. In addition, we expected faster RTs on repeated-emotion trials, for angry targets but nor for neutral targets, and only with upright faces (Lamy et al., 2008a). Of main interest was whether intertrial priming would improve search efficiency, that is, reduce search slopes.

### Methods

#### Subjects

Subjects were 34 Tel-Aviv university undergraduate students (18 females) who participated in the experiment for course credit. All reported having corrected-to-normal visual acuity.

#### Apparatus

Displays were generated by an Intel Pentium 4 computer attached to a 17″ CRT monitor, using 640 × 480 resolution graphics mode. Responses were collected via the computer keyboard. A chin-rest was used to set viewing distance at 50 cm from the monitor.

#### Stimuli

The fixation display was a gray plus sign (0.16 × 0.16°) in the center of a black background. The face stimuli were photographs of 8 different Caucasian individuals (4 males, 4 females) selected from the MacArthur battery of facial expressions stimuli (NimStim stimulus set: http://www.macbrain.org/resources.htm), with their mouths open. Each individual displayed either a neutral or an angry expression. Thus, the stimulus set consisted of 16 different pictures. All pictures were gray-scaled (8 bits). Mean luminance and contrast were matched between the pictures of the two different emotions of each individual.

Examples of the stimulus displays are presented in Figure 1. Each stimulus display consisted of the fixation display with either three or six faces of the same individual randomly scattered among the 8 peripheral positions of a 3 × 3 imaginary grid. Each cell subtended 5.1° in side. Each face was centered in its cell with a jitter of ±0.15° of visual angle, to prevent collinearities. In the target-absent condition, all three or six faces displayed the same emotional expression, either angry (all-angry condition) or neutral (all-neutral condition). In the target-present condition, one of the three or six faces displayed a different emotion, that is, there was either an angry face among neutral faces (angry-target condition), or a neutral face among angry faces (Neutral-target condition). For half of the subjects, all displays contained faces in their upright position, whereas for the remaining half all displays contained faces that were inverted upside down.

#### Procedure

Each trial began with the presentation of the fixation display for 500 ms. The stimulus display immediately followed and remained visible for 2500 ms or until response. The next trial began after 500 ms. Half of the subjects were instructed to detect the presence of a target by pressing “3” with their right index finger if one face was different from the others (target-present response) and “z” with their left index finger if all faces were identical (target-absent response). The remaining subjects were assigned the opposite key-to-response mapping for counterbalancing purposes. The experimenter did not refer to the fact that the target differed from non-targets by its emotional expression. Subjects were instructed to respond as quickly as possible, while maintaining high accuracy. Error trials were followed by a 500-msec feedback beep. Eye movements were not monitored, but subjects were explicitly requested to maintain fixation throughout each trial.

#### Design

A mixed design was used with face orientation (upright vs. inverted) as a between-subjects factor and target presence (present vs. absent), set size (3 vs. 6) and emotion (angry vs. neutral) as within-subject factors. On each trial, each face identity was equally likely to appear. On target-present-trials, the target was equally likely to appear in any of the eight possible locations. Set size (three vs. six), target presence (Target-present vs. Target-absent) and emotion (Angry vs. Neutral) were randomly mixed. The experiment began with one block of 50 practice trials, followed by 1024 trials divided into 8 blocks of 128 trials each.

### Results

Because examination of target repetition can be conducted only on target-present trials that are preceded by target-present trials, whereas search asymmetry can be examined using all trials, these effects were examined separately. In all RT analyses, error trials (6.4% with upright faces and 7.6% with inverted faces) were excluded and so were RT-outlier trials. Here as well as in the next experiment, these were defined as trials for which the reaction time exceeded the mean of its condition cell by more than 2.5 standard deviations.

#### Search asymmetry

Mean RTs and accuracy on target-present and on target-absent trials are presented in Table 1. Outlier trials (1.4%) were excluded from analysis. Upright and inverted face conditions were analyzed in separate analyses of variance (ANOVAs) with target presence (present vs. absent), distractor emotion (angry vs. neutral) and set size (3 vs. 6) as within-subject factors.

**Table 1:** **Mean reaction times (in milliseconds) and percentage of errors by conditions of target presence (present vs. absent), distractor on trial *n* (angry vs. neutral), feature repetition relative to trial *n-1* (repeated vs. switched) and set size (3 vs. 6) in Experiment 1, separately for upright faces and for inverted faces**.

	**Distractor on trial *n***	**Relative to trial *n-1***	**Set size 3**				**Set size 6**			
			**Mean RT**		**Mean % of errors**		**Mean RT**		**Mean % of errors**	
**UPRIGHT FACES**										
Target absent	Neutral	–	988	(34)	2.8%	(0.7%)	1168	(56)	2.1%	(0.5%)
	Angry	–	1040	(40)	5.7%	(0.7%)	1289	(69)	4.0%	(0.7%)
Target present	Neutral	Switched	1060	(48)	7.2%	(1.7%)	1147	(49)	8.7%	(1.6%)
		Repeated	1014	(37)	5.0%	(1.6%)	1095	(49)	9.2%	(1.7%)
	Angry	Switched	1056	(40)	8.3%	(1.8%)	1224	(54)	12.8%	(1.7%)
		Repeated	1067	(38)	8.2%	(1.7%)	1218	(47)	10.7%	(1.4%)
**INVERTED FACES**										
Target absent	Neutral	–	1035	(40)	4.7%	(0.9%)	1160	(46)	2.8%	(0.7%)
	Angry	–	1071	(48)	8.7%	(1.5%)	1265	(62)	6.2%	(1.0%)
Target present	Neutral	Switched	1073	(34)	9.7%	(2.5%)	1195	(57)	15.3%	(2.7%)
		Repeated	1098	(51)	8.2%	(1.5%)	1191	(48)	12.4%	(1.8%)
	Angry	Switched	1085	(46)	8.4%	(1.9%)	1275	(54)	16.1%	(3.2%)
		Repeated	1107	(51)	10.1%	(2.6%)	1205	(48)	13.2%	(2.7%)

*Standard errors are presented in parentheses*.

***Reactions times***. With upright faces, all main effects were significant, indicating that target-present trials were faster than target-absent trials, *F*_(1, 16)_ = 4.89, *p* = 0.042, neutral-distractor trials were faster than angry-distractor trials, *F*_(1, 16)_ = 17.97, *p* < 0.0006, and 3-item display trials were faster than 6-item display trials, *F*_(1, 16)_ = 80.70, *p* < 0.0001. The interaction between target presence and set size was significant, *F*_(1, 16)_ = 19.27, *p* < 0.0005, with larger search slopes when the target was absent than when it was present.

**Figure 2:**
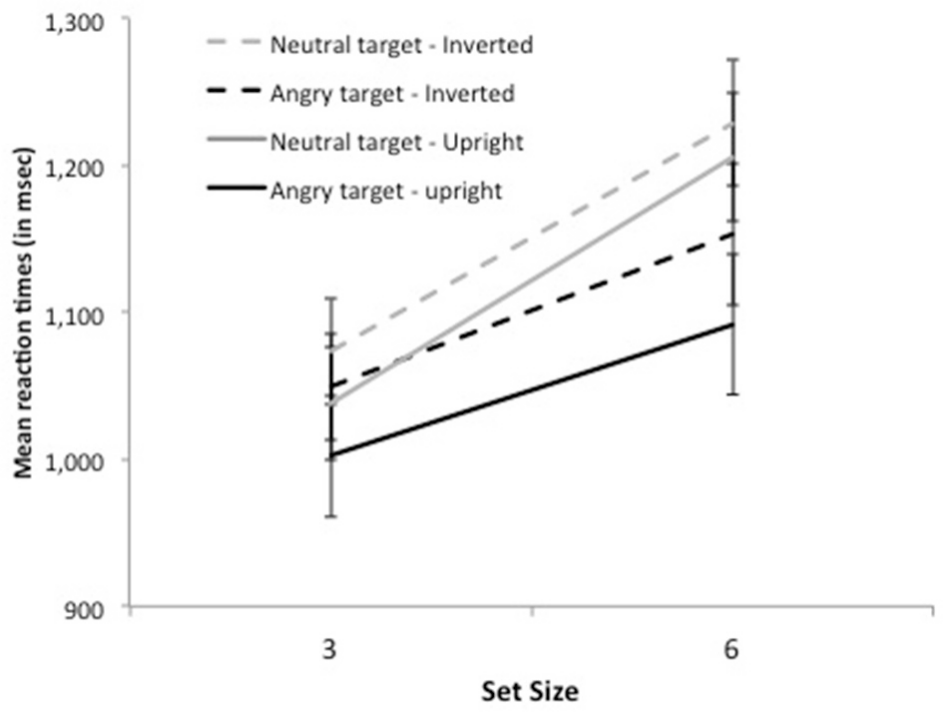
**Mean reaction times on target-present trials for set size 3 and 6 as a function of target emotion (angry or neutral) and face orientation (upright or inverted) in Experiment 1**.

The significant interaction between distractor emotion and display size, *F*_(1, 16)_ = 19.63, *p* < 0.0004, revealed the pattern characteristic of asymmetric search (Figure 2). On target-present trials, RTs were faster by 74 ms when the target was an angry face than when it was a neutral face, *F*_(1, 16)_ = 31.98, *p* < 0.0001, Cohen's *d* = 0.41, and search slopes were shallower, 35 ms/item, *F*_(1, 16)_ = 16.02, *p* < 0.0001, Cohen's *d* = 0.55 vs. 56 ms/item, *F*_(1, 16)_ = 32.79, *p* < 0.0001, Cohen's *d* = 0.84, respectively, as confirmed by the significant interaction between target emotion and set size, *F*_(1, 16)_ = 24.43, *p* < 0.0001. Likewise, on target-absent trials, RTs were faster by 86 ms on all-neutral than on all-angry displays *F*_(1, 16)_ = 9.28, *p* < 0.008, Cohen's *d* = 0.40, and search slopes were shallower, 60 ms/item, *F*_(1, 16)_ = 52.95, *p* < 0.0001, Cohen's *d* = 0.94 vs. 83 ms/item, *F*_(1, 16)_ = 55.97, *p* < 0.0001, Cohen's *d* = 1.07, respectively, as confirmed by the significant interaction between distractor emotion and set size, *F*_(1, 16)_ = 9.19, *p* < 0.008. No other effect was significant, all *F*s < 1.

The pattern of results was similar with inverted faces. The main effects of set size and distractor emotion were significant, *F*_(1, 16)_ = 86.48, *p* < 0.0001 and *F*_(1, 16)_ = 35.03, *p* < 0.0001, respectively. Neither the interaction between target presence and distractor emotion nor that between target presence and set size reached significance, *F*_(1, 16)_ = 3.29, *p* > 0.08 and *F*_(1, 16)_ = 2.36, *p* > 0.14, respectively. Again, the interaction between distractor emotion and display size was significant, *F*_(1, 16)_ = 11.79, *p* < 0.004. On target-present trials, RTs were faster by 50 ms when the target was an angry face than when it was a neutral face, *F*_(1, 16)_ = 39.11, *p* < 0.0001, Cohen's *d* = 0.62, and search slopes were shallower, 34 ms/item, *F*_(1, 16)_ = 9.58, *p* < 0.007, Cohen's *d* = 0.55 vs. 52 ms/item, *F*_(1, 16)_ = 31.06, *p* < 0.0001, Cohen's *d* = 0.95, respectively, as confirmed by the significant interaction between target emotion and set size, *F*_(1, 16)_ = 11.33, *p* < 0.004. Likewise, on target-absent trials, RTs were faster by 70 ms on all-neutral than on all-angry displays *F*_(1, 16)_ = 20.91, *p* < 0.0001, Cohen's *d* = 0.32, and search slopes were shallower, 42 ms/item, *F*_(1, 16)_ = 37.50, *p* < 0.0001, Cohen's *d* = 0.72 vs. 64 ms/item, *F*_(1, 16)_ = 43.16, *p* < 0.0001, Cohen's *d* = 0.85, respectively, as confirmed by the significant interaction between distractor emotion and set size, *F*_(1, 16)_ = 6.20, *p* < 0.03.

***Accuracy***. With upright faces, all main effects were significant, with more errors on target-present than on target-absent trials, *F*_(1, 16)_ = 56.24, *p* < 0.0001, on angry- than on neutral-distractor face trials, *F*_(1, 16)_ = 25.82, *p* < 0.0001, and in the 6- than in the 3-item condition, *F*_(1, 16)_ = 7.76, *p* < 0.02. The interaction between target presence and set size was significant, *F*_(1, 16)_ = 24.05, *p* < 0.0001. It was modulated by a significant 3-way interaction, *F*_(1, 16)_ = 5.80, *p* < 0.03 indicating that while on target-present trials, search slopes tended to more positive for angry-than for neutral-distractor trials, *p* > 0.12, on target-absent trials they tended to be more negative, *p* > 0.13. There was no other significant effect, all *F*s < 1.

With inverted faces, all main effects were significant, paralleling the findings with upright faces, *F*_(1, 16)_ = 48.20, *p* < 0.0001, *F*_(1, 16)_ = 7.48, *p* < 0.02 and *F*_(1, 16)_ = 23.86, *p* < 0.0002 for target presence, distractor emotion and set size, respectively. The interaction between target presence and set size was significant, *F*_(1, 16)_ = 44.78, *p* < 0.0001, with more errors with 6- than on 3-item displays on target-present trials, but the opposite on target-absent trials. The interaction between target presence and distractor emotion was also significant, *F*_(1, 16)_ = 4.64, *p* < 0.05, indicating that participants made more errors on angry-distractor trials only on target-absent but not on target-present trials. No other effect reached significance, all *ps > 0.15*.

#### Emotional inter-trial priming

Trial sequences that involved the same face identity on successive trials (11% of the trials) were excluded from analysis in order to avoid contamination of the effect of emotion repetition by potential effects of physical features repetition. The pattern of results was similar when these trials were included.

An ANOVA with target emotion (angry vs. neutral), emotion repetition (repeated vs. switched) and set size (3 vs. 6) as within-subject factors was conducted on target-present trials that were preceded by a target-present trial, separately for each condition of face orientation. Outlier trials (1.2%) were removed from analysis.

**Figure 3:**
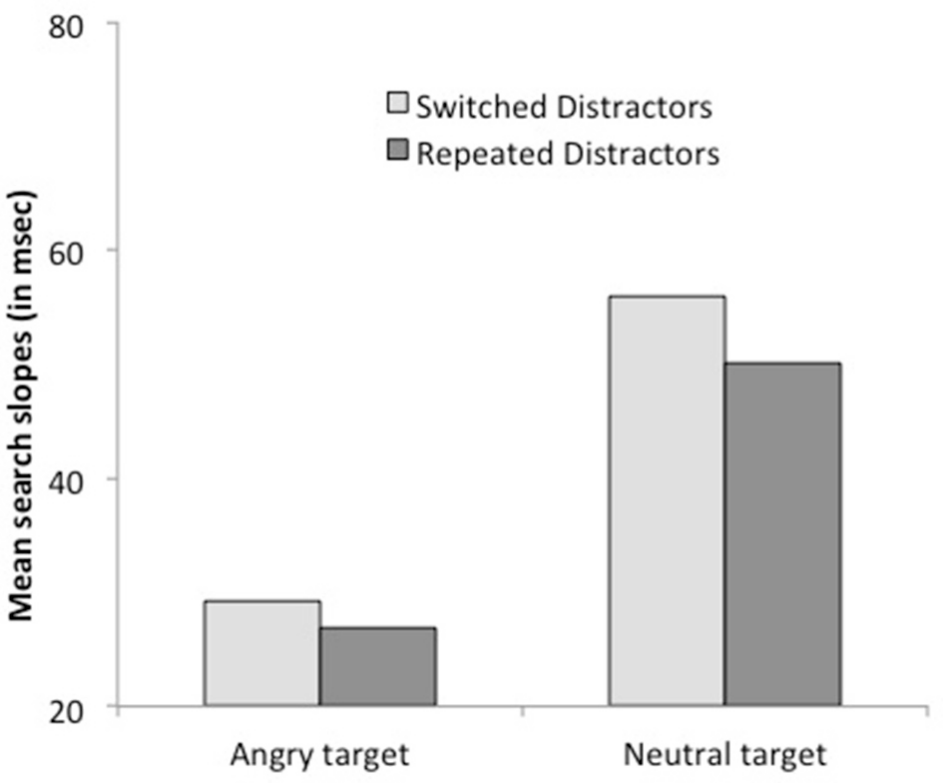
**Mean search slopes (mean additional reaction time for each added distractor) on target-present trials preceded by a target-present trial as a function of target emotion (angry or neutral) and target repetition (repeated or switched) in Experiment 1**.

***Reaction times***. With upright faces, subjects were faster when the target repeated on successive trials, *F*_(1, 16)_ = 6.54, *p* < 0.03. This effect interacted with target emotion, *F*_(1, 16)_ = 4.88, *p* < 0.05: it was larger when the target was an angry face, 49 ms, *F*_(1, 16)_ = 11.23, *p* < 0.005, Cohen's *d* = 0.32 than when it was a neutral face, 3 ms, *F* < 1, Cohen's *d* = 0.02. The target repetition effect did not interact with set size (see Figure 3). Search slopes were equally large whether the target repeated or switched on successive trials, both for angry targets, 27 vs. 29 ms/item, respectively and with neutral targets, 50 ms vs. 55 ms, respectively, both *Fs < 1*. Additional analyses based on the BIC (Bayesian information criterion) estimate (Masson, 2011) revealed posterior probabilities of 94.2 and 87.2% for a null interaction between target repetition and set size, for angry and neutral targets, respectively. These outcomes provide positive evidence for the notion that target repetition does not improve search efficiency (Raftery, 1995).

With inverted faces there was no effect of emotion repetition, *F* < 1 and none of the interactions involving this factor was significant, all *p*s > 0.1.

***Accuracy***. None of the effects involving emotion repetition was significant with either upright or inverted faces, all *p*s > 0.2.

### Discussion

In this experiment, search for an emotional singleton showed the asymmetric pattern characteristic of the face-in-the-crowd phenomenon. Search was faster and search slopes were half as steep (35 vs. 56 ms per item) when the target was an angry face than when it was a neutral face. Notably, this pattern was closely replicated when the faces were inverted instead of upright, a finding that will be further elaborated upon in the general discussion. In addition, we replicated the emotional intertrial priming findings reported earlier (Lamy et al., 2008b). Search performance was faster when the target's facial expression repeated on consecutive trials than when it switched. This effect occurred only for angry targets and was completely abolished when the faces were inverted.

Most crucially, although search slopes were substantial on angry-target trials, repetition of the target did not increase search efficiency. These results suggest that inter-trial priming do not affect preattentive allocation of attentional priority.

## Experiment 2

The objective of Experiment 2 was to determine whether the findings of Experiment 1 could be replicated with simple stimuli, for which search asymmetry has been demonstrated many times, namely Os and Qs (e.g., Treisman and Souther, 1985; Saiki et al., 2005). This experiment was similar to Experiment 1 except that the target was either an O among Qs or a Q among Os. Display size was again either 3 and 6 and the target was equally likely to be present or absent.

### Methods

#### Subjects

Subjects were 14 Tel-Aviv University undergraduate students who participated in the experiment for course credit. All reported having normal or corrected visual acuity and normal color vision.

#### Apparatus

Displays were generated by an Intel Pentium 4 computer attached to a 17″ CRT monitor, using 640 × 480 resolution graphics mode. Responses were collected via the computer keyboard. A chin-rest was used to set viewing distance at 60 cm from the monitor.

#### Stimuli

The fixation display was a plus sign (1.14 × 1.14°. of visual angle) in the center of the screen. Search items were Os (circles with a diameter of 0.7° of visual angle) and Qs (similar to the Os except for the addition of a 0.35° vertical bar centered on the lower point of the Os, such that half of the bar was inside the circle and the other half outside of it). Each search display contained either 3 or 6 items. The shapes appeared at random locations within an imaginary 6 × 8 matrix centered at fixation. Each cell subtended 1.5° in side and each shape was centered inside its cell with a random jitter of −0.15°, 0 or 0.15°. On target-present trials, one item was unique, either an O among Qs, or a Q among Os. On target-absent trials, all the items in the display were the same, either all Os or all Qs. All stimuli were drawn with a gray 1-pixel stroke on a black background.

#### Procedure and design

The procedure and design were similar to those of Experiment 1 except for the following changes. The stimulus display remained visible until response or for 2000 ms. The experiment began with one practice block including 16 trials and followed by 16 experimental blocks of 50 trials each. All variables were manipulated within subjects, and were equiprobable and randomly mixed.

### Results and discussion

#### Search asymmetry

In all RT analyses, error trials (3.6% of all trials) were removed from analysis. Mean RTs and accuracy on target-present and on target-absent trials are presented in Table 2. Outlier trials (1.2%) were removed from analysis.

**Table 2:** **Mean reaction times (in milliseconds) and percentage of errors by conditions of target presence (present vs. absent), distractor on trial *n* (Q vs. O), feature repetition relative to trial *n-1* (repeated vs. switched) and set size (3 vs. 6) in Experiment 2**.

	**Distractor on trial *n***	**Relative to trial *n-1***	**Set size 3**				**Set size 6**			
Target absent	O	–	613	(24)	2.2%	(0.7%)	620	(23)	2.2%	(0.8%)
	Q	–	731	(22)	5.9%	(1.2%)	783	(26)	3.5%	(0.9%)
Target present	O	Switched	679	(35)	3.2%	(1.3%)	689	(26)	2.1%	(0.9%)
		Repeated	625	(27)	2.9%	(1.1%)	654	(30)	2.7%	(1.5%)
	Q	Switched	664	(30)	2.4%	(1.1%)	734	(26)	5.3%	(1.8%)
		Repeated	660	(33)	2.2%	(0.8%)	717	(30)	8.1%	(2.2%)

*Standard errors are presented in parentheses*.

**Figure 4:**
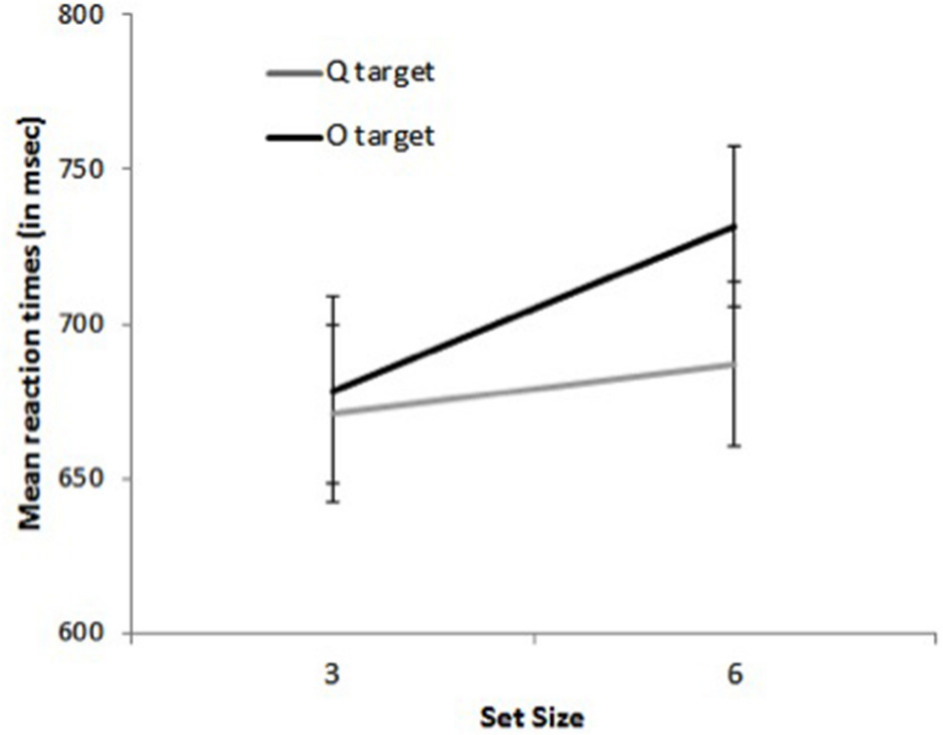
**Mean reaction times on target-present trials for set size 3 and 6 as a function of target shape (O and Q) in Experiment 2**.

***Reactions times***. The main effects of distractor shape and display size were significant, with faster RTs for O than for Q distractors, *F*_(1, 13)_ = 100.45, *p* < 0.0001, and for 3- than for 6-item displays, *F*_(1, 13)_ = 54.80, *p* < 0.0001. The interaction between distractor shape and target presence was significant, *F*_(1, 13)_ = 60.39, *p* < 0.0001, with a larger effect of distractor shape for target-absent than on target-present trials, 140 vs. 29 ms. The results again showed the pattern characteristic of asymmetric search (Figure 4): on target-present trials, RTs were faster by 29 ms when the target was a Q than when it was an O, *F*_(1, 13)_ = 40.30, *p* < 0.0001, Cohen's *d* = 0.28, and search slopes were shallower, 5.1 ms/item, *F*_(1, 13)_ = 4.61, *p* < 0.06, Cohen's *d* = 0.13 vs. 16.1 ms/item, *F*_(1, 13)_ = 26.90, *p* < 0.0002, Cohen's *d* = 0.49, respectively, as confirmed by the significant interaction between target letter and set size, *F*_(1, 13)_ = 33.02, *p* < 0.0001. Likewise, on target-absent trials, RTs were faster by 140 ms on all-O than on all-Q displays *F*_(1, 13)_ = 93.51, *p* < 0.0001, Cohen's *d* = 1.56, and search slopes were shallower, 2 ms/item, *F*_(1, 16)_ = 2.25, *p* > 0.15, Cohen's *d* = 0.08 vs. 28 ms/item, *F*_(1, 13)_ = 21.67, *p* < 0.0005, Cohen's *d* = 0.58, respectively, as confirmed by the significant interaction between distractor emotion and set size, *F*_(1, 13)_ = 13.83, *p* < 0.003. No other effect was significant, all *F*s < 1.

***Accuracy***. Mirroring the RT data, error rates were lower when the target was a Q than when it was an O, *F*_(1, 13)_ = 5.14, *p* < 0.05, and the set size effect was smaller, 0.5%, *F* < 1 vs. 2.9%, *F*_(1, 13)_ = 8.54, *p* < 0.02, respectively, as confirmed by the significant interaction between target letter and set size, *F*_(1, 13)_ = 6.63, *p* < 0.03.

#### Intertrial priming

Only target-present trials preceded by target-present trials were entered in these analyses. Outlier trials (1.0% of the trials) were excluded from the analyses.

**Figure 5:**
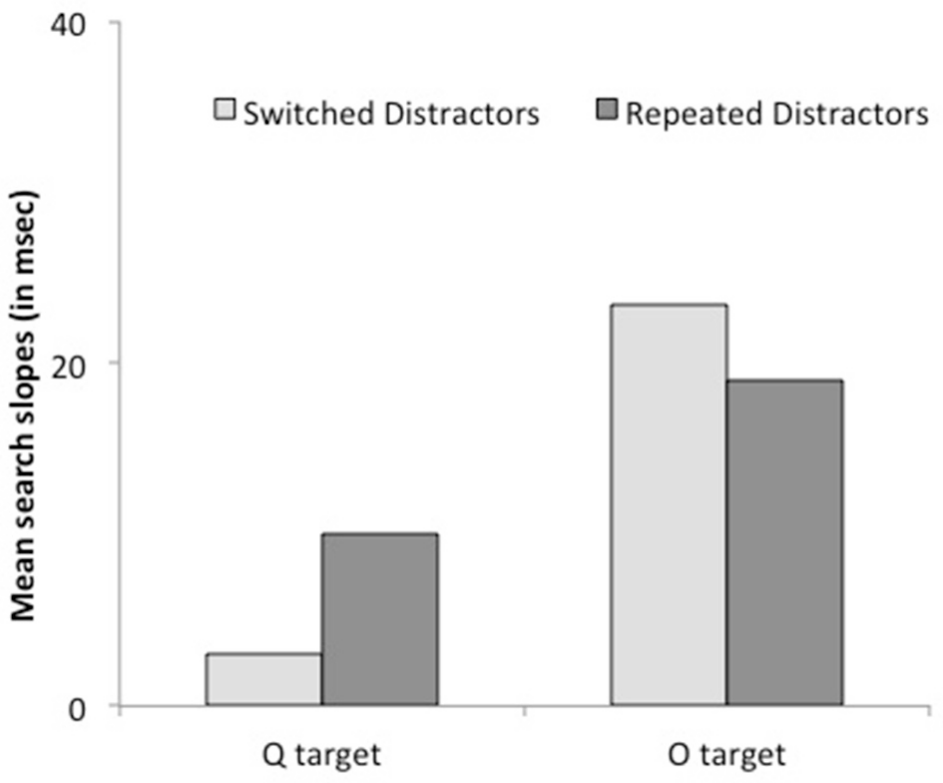
**Mean search slopes (mean additional reaction time for each added distractor) on target-present trials preceded by a target-present trial as a function of target shape (O or Q) and target repetition (repeated or switched) in Experiment 2**.

***Reaction times***. Subjects were faster when the target repeated on successive trials, *F*_(1, 13)_ = 8.39, *p* < 0.02. This effect interacted with target letter condition, *F*_(1, 13)_ = 5.86, *p* = 0.03: it was larger when the target was a Q, 55 ms, *F*_(1, 13)_ = 17.24, *p* < 0.002, Cohen's *d* = 0.41 than when it was an O, 6 ms, *F* < 1, Cohen's *d* = 0.05. Crucially, there was no interaction between set size and target repetition, *F* = 0.00: search slopes were similar whether the target repeated or did not, 14.4 vs. 14.5 ms/item. The 3-way interaction approached significance, *F*_(1, 13)_ = 3.40, *p* < 0.09, yet further analyses revealed that it resulted from non-significant trends in opposite directions for the two target-letter conditions, both *p*s >0.2 (see Figure 5). Additional analyses based on the BIC (Bayesian information criterion) estimate (Masson, 2011) revealed posterior probabilities of 84.6 and 76.5% for a null interaction between target repetition and set size, for Q and O targets, respectively. These outcomes provide positive evidence for the notion that target repetition does not improve search efficiency (Raftery, 1995).

***Accuracy***. No effect involving target repetition was significant, all *p*s > 0.12, nor was any effect involving distractor repetition, all *p*s > 0.24. Thus, speed-accuracy trade-off was not a concern.

The findings of Experiment 1 were fully replicated. The search showed the characteristic asymmetric pattern, with faster RTs and shallower search slopes for Q than for O targets, respectively. Intertrial priming was observed and also showed an asymmetrical pattern. It was significant only for the most salient target, Q and did not interact with set size. Taken together, our findings reflect a general pattern observed not only with complex naturalistic stimuli but also with simple shapes, during asymmetric search.

## General discussion

### Intertrial priming and attentional priority

The main objective of this study was to test whether intertrial priming affects attentional priority allocation in visual search. If it does, then target repetition should reduce search slopes during serial search. The results were clear: repetition of the target did not make the search more efficient, neither in fairly serial search (>50 ms/item) nor in moderately serial search (<20 ms/item) and neither with complex stimuli (veridical angry and neutral faces) nor with simple shapes (Os and Qs). Thus, the results support the view that intertrial priming does not affect preattentive processing during which attentional priorities are ranked, but later perceptual processes that occur during the allocation of focal attention (e.g., Yashar and Lamy, 2010; Wan and Lleras, 2010).

Two findings of the present study seem to stand in contradiction with previous reports. First, we found intertrial priming effects in a detection task (see also Olivers and Meeter, 2006; Lamy et al., 2008b), but previously claimed that these can be observed only when the search task requires focused attention (Yashar and Lamy, 2010; see also Wan and Lleras, 2010). However, the need for focused attention in visual search depends on target-distractor discriminability rather than on the task *per se*. With highly discriminable features, the target can be detected with distributed attention, whereas with low target-distractor discriminability, which is typically associated with positive search slopes (Duncan and Humphreys, 1989), detection also may require focused attention (see Feldmann-Wüstefeld et al., 2011, for electrophysiological evidence that detection of an angry target among neutral faces requires focused attention).

The second finding is that the easier search yielded the larger intertrial priming effects (see also Lamy et al., 2008b)^1^: repetition effects were observed only with angry-face and Q targets, which yielded faster and more efficient search than neutral-face and O targets. Previous studies that investigated the influence of search difficulty on intertrial priming reported the opposite pattern of results (e.g., Meeter and Olivers, 2006; Lamy et al., 2011). However, the discrepant findings emerged from markedly different search difficulty manipulations. In previous studies (Meeter and Olivers, 2006; Lamy et al., 2011), the local contrast at the target location was smaller in the difficult than in the easy search condition and the two conditions of search difficulty involved two different pairs of stimuli. In contrast, here, we used an asymmetric search task, such that the local contrast at the target location remained unchanged in the two conditions of search difficulty. In addition, these involved the same pair of stimuli and the difference between the two conditions hinged on which of these two stimuli was the target. It is thus likely that not search difficulty *per se* but the asymmetry of the search drives the asymmetry in intertrial priming, as discussed below.

### Inter-trial priming and search asymmetry

Our second objective was to investigate intertrial priming asymmetry. We showed that it is a general phenomenon: both with complex stimuli (replicating Lamy et al.'s, 2008b findings) and with simple letters, we observed repetition effects only with the more salient targets. Intriguingly, however, asymmetric search was not necessarily associated with asymmetric priming. In Experiment 1, when the faces were inverted, search was asymmetric (with markedly shallower search slopes when the target was angry than when it was neutral), yet priming was equally absent for both target conditions. What, then, may drive priming asymmetry?

The effects of face inversion in Experiment 1 suggest that different features drove search asymmetry and intertrial priming asymmetry. Specifically, one feature increased search efficiency irrespective of whether the faces were upright or inverted: the feature that distinguished the angry from the neutral version of the same face made it more salient (as evidenced by the shallower search slopes) and was likely to be a low-level feature rather than a configural property of the emotional face stimuli because its effects were unaffected by face inversion (see Tanaka and Farah, 1993; Maurer et al., 2002; for detailed explanations of inferences from the face inversion manipulation and see Horstmann and Bauland, 2006, for a previous report of search asymmetry with inverted emotional faces). In contrast, the feature that drove intertrial priming and its asymmetry (i.e., priming with angry- but not with neutral-face targets) emerged from a higher-level, configural, holistic representation that was disrupted when the faces were inverted.

Consistent with conjecture, note that the intertrial effect reported here relied on features that were common to *different* individuals displaying an angry expression, whereas search asymmetry effects relied on a property that distinguished the angry expression of one individual from the neutral expression of the *same* individual. If one assumes that a collection of different features characterize a given specific angry face to varying degrees (e.g., the curve of the mouth, the orientation of the eyebrows, the wideness of the eyes, etc…), then these features do not necessarily repeat on successive trials portraying different individuals: only the higher-level emotional content of the face does, and drives only intertrial priming.

We suggest that the feature associated with successful search on the previous trial must be consciously detected in order to be encoded in short-term visual memory and consequently speed the deployment of focal attention when consciously detected in the target during the current trial—thereby yielding intertrial priming (see Peremen et al., 2013 for a demonstration that intertrial priming is contingent on conscious detection of the target). Furthermore, to explain the priming asymmetry observed here, we rely on Treisman and Souther's (1985) suggestion that a critical aspect of asymmetric search is that one target has a unique distinguishing feature, whereas the other target is distinguished only by the absence of a feature that is present in all the distractors. Specifically, we propose that conscious detection of the presence of a feature but not of its absence is associated with priming effects. Accordingly, priming was absent with inverted faces because (1) the salient physical feature underlying the search asymmetry did not repeat from one trial to the next (as the photographed individuals were different on consecutive trials) and (2) the emotion conveyed by the inverted angry face was not perceived. In contrast, with upright angry-face targets, as well as with Q targets, the added feature that distinguished the target from the distractors (the angry emotion and the oblique bar of the Q) was perceived and was therefore associated with priming.

## Conclusions

Our findings suggest that intertrial priming does not improve search efficiency in asymmetric search and therefore takes effect after attentional priorities have been assigned—but it will be important to examine whether these findings generalize to serial searches that are not asymmetric. In addition, we showed that asymmetric search is associated with asymmetric priming effects and suggested that priming occurs only when the target is characterized by an additional feature that is consciously perceived, and not when it is characterized by this feature's absence.

## Author's note

Support was provided by the Israel Science Foundation (ISF) Grant No. 1475/12 and the Binational Science Foundation (BSF) Grant No. 2009425 to Dominique Lamy We thank Amos, Roey Rudoy and Dror Cohen for their help in running the experiments.

### Conflict of interest statement

The authors declare that the research was conducted in the absence of any commercial or financial relationships that could be construed as a potential conflict of interest.

## Footnotes

^1^The idea that a target can be selected and responded to without focal attention is widely accepted (e.g., Bravo and Nakayama, 1992; Yashar and Lamy, 2010); yet it was recently challenged by Theeuwes et al. (2008).

^2^It is noteworthy that in the same study but relying on eye movement data, Becker (2008) showed that intertrial target repetition decreased the number of nontarget fixations before target selection, suggesting that intertrial priming did increase the target's attentional priority. However, this effect relied on an extremely small number of erroneous-fixation trials (it ranged 0.2 to 0.9, indicating that on average, each observer made less than one erroneous fixation in each experimental condition). Thus, taking into account that in the same study, intertrial priming did not reduce distractor interference, these findings provide only tenuous support for the claim that intertrial priming increases target salience.
